# Design and Deployment of a Pediatric Cardiac Arrest Surveillance System

**DOI:** 10.1155/2018/9187962

**Published:** 2018-05-09

**Authors:** Jordan Michel Duval-Arnould, Heather Marie Newton, Leann McNamara, Branden Michael Engorn, Kareen Jones, Meghan Bernier, Pamela Dodge, Cheryl Salamone, Utpal Bhalala, Justin M. Jeffers, Lilly Engineer, Marie Diener-West, Elizabeth Anne Hunt

**Affiliations:** ^1^Division of Health Sciences Informatics, Department of Anesthesiology and Critical Care Medicine, School of Medicine, The Johns Hopkins University, Baltimore, MD, USA; ^2^Department of Occupational Health, The Johns Hopkins Hospital, Baltimore, MD, USA; ^3^Department of Pharmacy, The Johns Hopkins Hospital, Baltimore, MD, USA; ^4^Department of Anesthesiology and Critical Care Medicine and Department of Pediatrics, School of Medicine, The Johns Hopkins University, Baltimore, MD, USA; ^5^Department of Anesthesiology and Critical Care Medicine, School of Medicine, The Johns Hopkins University, Baltimore, MD, USA; ^6^Department of Pediatrics, The Johns Hopkins Hospital, Baltimore, MD, USA; ^7^Neonatology Respiratory Therapy, The Johns Hopkins Hospital, Baltimore, MD, USA; ^8^Department of Biostatistics, Johns Hopkins University Bloomberg School of Public Health, Baltimore, MD, USA

## Abstract

**Objective:**

We aimed to increase detection of pediatric cardiopulmonary resuscitation (CPR) events and collection of physiologic and performance data for use in quality improvement (QI) efforts.

**Materials and Methods:**

We developed a workflow-driven surveillance system that leveraged organizational information technology systems to trigger CPR detection and analysis processes. We characterized detection by notification source, type, location, and year, and compared it to previous methods of detection.

**Results:**

From 1/1/2013 through 12/31/2015, there were 2,986 unique notifications associated with 2,145 events, 317 requiring CPR. PICU and PEDS-ED accounted for 65% of CPR events, whereas floor care areas were responsible for only 3% of events. 100% of PEDS-OR and >70% of PICU CPR events would not have been included in QI efforts. Performance data from both defibrillator and bedside monitor increased annually. (2013: 1%; 2014: 18%; 2015: 27%).

**Discussion:**

After deployment of this system, detection has increased ∼9-fold and performance data collection increased annually. Had the system not been deployed, 100% of PEDS-OR and 50–70% of PICU, NICU, and PEDS-ED events would have been missed.

**Conclusion:**

By leveraging hospital information technology and medical device data, identification of pediatric cardiac arrest with an associated increased capture in the proportion of objective performance data is possible.

## 1. Background and Significance

Cardiac arrest (CA) is a fatal medical condition as well as a significant public health challenge. The most recent estimates suggest that in the United States (U.S.), approximately 5,00,000 adults and children experience a cardiac arrest annually; globally the number is in the millions. In the U.S., survival from cardiac arrest is less than 15% [1–5]. Though less common in children than adults, pediatric sudden cardiac arrest incidence, case fatality, and years of potential life lost are all significant.

Literature published at the turn of the century suggests that pediatric cardiac arrests occur in 0.7% to 3% of pediatric hospital admissions and 1.8% to 5.5% of pediatric intensive care unit (PICU) admissions [6–9]. Nation-wide 4,000 pediatric in-hospital cardiac arrests (IHCAs) per year require at least two minutes of CPR [10]. A recent multicenter study suggests that less than 25% pediatric IHCAs require less than 2 minutes [11]; evaluation of 5 years of epidemiologic data from this institution suggests this could be as high as 37% (165/446). The PICU estimates suggest at least one cardiac arrest per 100 admissions [12]. Recent estimates suggest that the number of annual PICU admissions is between 2,30,000 and 4,10,000, resulting in a possible annual range of cardiac arrests from 2,300 to 4,100 in PICUs in the U.S. [13, 14].

Pediatric in-patients are increasingly monitored by a variety of methods, along with regular and frequent interaction by a range of providers throughout the course of their care. Despite this high degree of electronic and human monitoring, when an IHCA occurs, awareness of the event may be limited to the primary team involved with the patient. Individuals who are distant from the event, whether it be geographically (the other side of the unit/building), temporally (the next day), or institutionally (another department), may be unaware that the event even occurred.

At this institution, measurement of the incidence of “true cardiac arrests” was a perpetual and particularly stubborn challenge, and accurate statistics were essentially nonexistent. Despite electronic health record (EHR) documentation, participation in a large cardiac arrest and CPR registry, dedicated resources to abstract and enter data, internal emergency response teams, bedside monitors and “code blue” buttons, surveillance of these events was limited due to the manual approach for event detection rather than the use of automated or semiautomated methods.

Identification of cardiac arrest events outside of the Pediatric Intensive Care Unit (PICU) usually took place when the Pediatric Rapid Response Team (RRT) was called. At this point, the event was documented on a standardized paper flow sheet and eventually handed to the organizational group responsible for event accounting. Pediatric cardiac arrest is rare in general care settings [15] and tends to occur less frequently on the wards versus other critical care locations such as the Pediatric Emergency Department (PEDS-ED), PICU, operating room (OR), and magnetic resonance imaging (MRI) procedures areas [16]. In these areas, the RRT is seldom activated, because the teams trained in acute pediatric resuscitation were already physically present. In the MRI procedure areas, it is standard practice for either a critical care nurse, and/or fellow, and/or respiratory staff to accompany the patients needing these procedures. It is also very common for an attending anesthesiologist to be present performing related tasks involved in the procedure, and thus the identification mechanism is often not triggered, given the availability of advanced practice providers and staff. Also, in most PICUs, PEDS-EDs, and ORs, there are unit-specific code alerts and code teams. This reduces the likelihood that these acute and critical care area events will be detected, documented, and discussed as part of evidence-based quality improvement initiatives at an institutional level. In order for all events to be captured, an active and reliable surveillance system is needed.

In April 2012, this institution's entire pediatric population was moved into a new clinical building. Despite the advanced technology present in the building, including integrated nurse call, code blue button, and electronic paging systems, there was no increase in the detection of CA events. These systems, among others, were not being leveraged to serve as notification sources of possible cardiac arrest for use as part of an active surveillance system. As a result, valuable data captured by advanced bedside monitors and smart defibrillators (those that display and record patient and performance data during CPR) were not being collected or evaluated and ultimately not being used to benefit providers or future patients.

This loss of data and underidentification of pediatric CA events represent significant missed opportunities for learning, performance improvement, and contribution to a larger body of scientific knowledge. These opportunities align directly with the National Academies' 2015 recommendations as described in “strategies to improve cardiac arrest survival” including the need for comprehensive surveillance, the need for robust data collection and dissemination, and improvement of the delivery of high-quality resuscitation [17].

## 2. Objective

The aim of this study is to increase the detection of pediatric CPR resuscitation events and collection of physiologic and performance data through the implementation of partially automated, workflow-driven CA surveillance system.

## 3. Materials and Methods

### 3.1. Process Flow: Resuscitation Event Analysis Clearinghouse (REACH) Surveillance System

**Figure 1:**
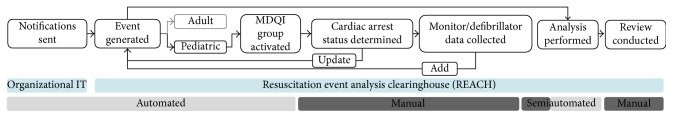
Conceptual model of pediatric cardiac arrest surveillance system process flow.

After IRB approval, existing information systems utilized on the medical campus, and specifically, at the onset of CA were identified as surveillance notification data sources. These systems were configured to automatically message the implemented computer-based surveillance system known by the acronym “REACH” each time they were activated, providing information regarding date, time, and location of event. The system attempted to capture any possible pediatric CA (defined as a child who received chest compressions and/or defibrillation). Logic was developed to screen notifications as they were added to the system by a combination of factors including geographic origin and message text; these were iteratively refined in order to automatically differentiate potential pediatric events versus those events as definitely adult in nature. This institution is not a free-standing pediatric facility but rather a children's center further integrated in a larger medical campus. Pediatric notifications were automatically disseminated to a 60 person multidisciplinary quality improvement (MDQI) group via email as a “potential CA.” Membership of the MDQI included representatives from all hospital care areas, and included residents, fellows, faculty, nurses, respiratory therapists, and pharmacists. The flow to this point was fully automated, standardized, and required no human intervention since it was driven by preexisting provider-based task workflow (Figure 1).

The manual part of the process began when the MDQI group received notifications and determination of CA status took place. When a true CA was verified, these events were designated as such in REACH and data collection of bedside monitor and smart defibrillator data was initiated. Notification, event, physiologic, and performance data were analyzed and made available for review through different organizational mechanisms. The technology acceptance model informed the overall design process [18]. We considered maximizing the perceived ease of use through the integration of preexisting workflows and organizational IT systems. We aimed to maximize the system's perceived usefulness by providing data in live-time to the group regarding the event status (i.e., improved situational awareness regarding acute events in the hospital) as well as postevent performance assessment based on data obtained upon successful completion of the detection process.

### 3.2. Design: Surveillance System Components

*(A) Organizational IT Systems*. It is used at the onset or during cardiac arrest and capable of sending email messages. The ability of every system identified to send email messages in the periarrest period largely drove the design decision to leverage email as the messaging protocol for notifications.  Appendix A.1 describes each system and configuration considerations necessary for integration.

*(B) Organizational Enterprise Email*. It receives messages from the organizational IT systems. Based on the sender, it flags messages as valid for consumption by polling service. It uses rule-based processing to identify likely pediatric-related notifications. It relays pediatric notifications to MDQI listserv.

*(C) Relational Database*. It stores the following: user information, notification, event, CPR, and various rules, preference, and usage data.

 
*(C1)*. It is automatically backed up fully every 24 hours and differentially every 1 hour.

*(D) System Logic and User Interface*. It provides management of notification, event, and monitor/smart defibrillator records and data analysis features.

*(E) Listserv*. It includes the members of the MDQI group. This list is the primary method by which cardiac arrest status communicated amongst the group once determined.

**Figure 2:**
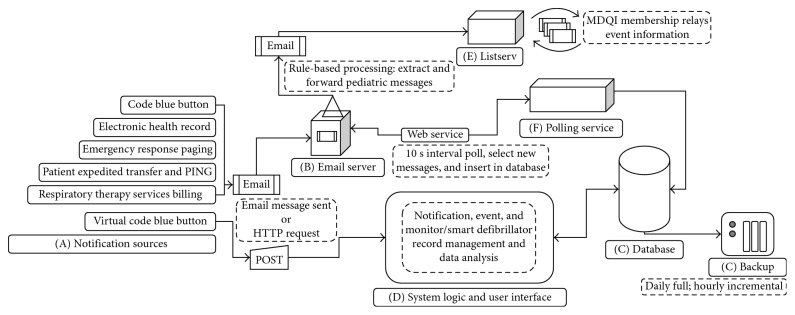
Pediatric cardiac arrest surveillance system data flow.

*(F) Polling Service*. It queries the organizational email, retrieves and extracts new notification message data, and standardizes and inserts in database (Figure 2).

### 3.3. Deployment

**Figure 3:**
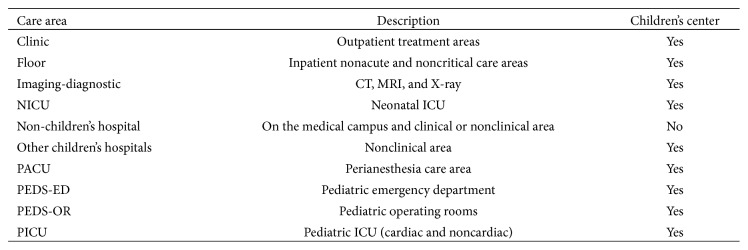
Notification and event location types.

The system was developed over a six-month period, tested for two months, and launched on January 1, 2013; reporting and analysis features were added in 2014. As notifications were generated and sent to the system, each was associated with an event; multiple notifications could be linked to the same event. An event location was associated with one of the 10 possible care areas (Figure 3) and designated as a CA event or not. For events that were designated CA, if smart defibrillator and/or bedside monitor data were collected these were added to the event record.

## 4. Results

### 4.1. Surveillance System Identification and Detection of Events

**Figure 4:**
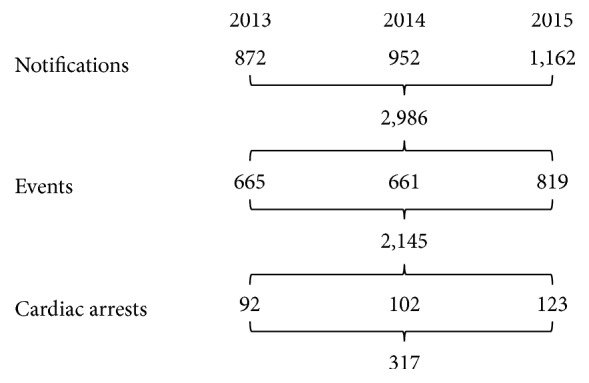
Aggregate and annual frequency of pediatric notifications, events, and cardiac arrests.

For the period 1/1/2013 through 12/31/2015, there were 2,986 unique notifications (i.e., the triggering of a notification source: Figure 2 “A” or Appendix A.1) associated with 2,145 events, of which 317 were designated as CA requiring chest compressions and/or defibrillation (Figure 4). There were 1.4 notifications per event (range: 1–7). Seventy percent (70%) of events had three or less notifications. Only three events had the maximum (seven) notifications observed; all were CA events. CA events made up approximately 15% of all events detected by the surveillance system.

### 4.2. Notifications, Events, and CA by Care Area

The PICU, floor, and PEDS-ED were the top three notification generators with 983, 1030, and 365, respectively (Table 1). These care areas also had the most events (PICU : 854, floor : 512, PEDS-ED : 315). The PICU and PEDS-ED accounted for 65% of all CA events, whereas floor care areas experienced 3% of all pediatric CA events.

Approximately 20% of PICU and PEDS-ED notifications and events were CA-related (Table 2). The proportion of PEDS-OR events and notifications that were CA-related was 32%, and the NICU and Imaging-diagnostic care areas had 30 and 37% of events being CA-related, respectively; 40% of notifications were CA-related for both areas.

### 4.3. Differences in Notifications per Event Given CA Event Status

When comparing the total number of notifications per event by CA status, there was a statistically significant difference between the two groups (CA: 1.6 versus non-CA: 1.4; *p* < 0.001; Wilcoxon rank-sum) (Table 3).

### 4.4. Surveillance Performance

Examining the type of notification sources associated with each CA event allowed for the determination of whether the event would have been detected had the surveillance system not been put in place (Table 4). Events were flagged as having been identified by any other source other than the EHR or the emergency response paging (methods available and utilized prior to the deployment of REACH). One-hundred percent of the PEDS-OR CA events would not have been detected and/or reported. Over 70% of PICU and approximately 50% of both PEDS-ED and NICU events would not have been detected and/or reported for use in QI efforts.

### 4.5. Smart Defibrillator and Bedside Monitor Data Collection

**Figure 5:**
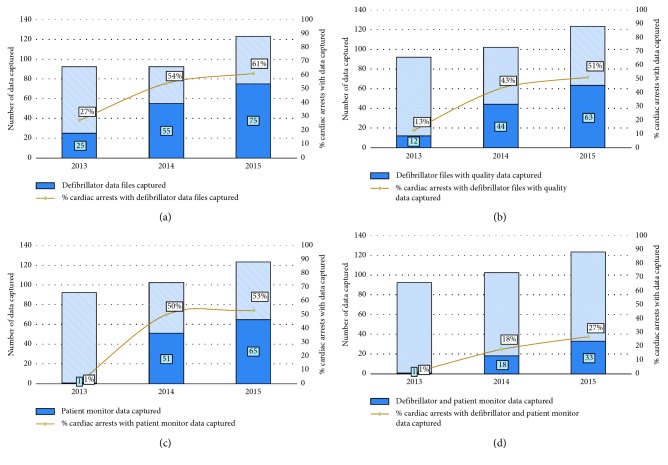
Proportion of CA events with smart defibrillator and bedside monitor data collected by year (2013–2015).

Over the study period, there was an increase in the proportion of CA events that had defibrillator records, defibrillator records with quality of CPR data, and bedside monitor data collected. More records were collected than had usable quality of CPR data. Defibrillator pads capable of measuring quality in patients smaller than 25 kg were not available until 2014, thus skewing these results. When evaluating events that had both usable CPR data from the defibrillator and bedside monitor data, this proportion increased from year to year but was still relatively low (2013: 1%; 2014: 18%; 2015: 27%). As pediatric CA events are detected, the REACH system triggers patient and performance data collection mechanisms used to drive weekly debriefing of events. Over the period, both the defibrillator and bedside monitor data collection increased and were used during debriefings. For 2014 and 2015, however, 16–20% of defibrillator records collected did not contain the quality of CPR data (Figure 5). Methods to collect bedside monitor were not available until the end of 2013. Both methods and processes were formalized in early 2014 resulting in approximately 50% of events having monitor data collected.

## 5. Discussion

In 2015, the National Academies described a framework for “improving patient outcomes from cardiac arrest.” This framework rests on a foundation of comprehensive surveillance and reporting underpinned by reliable and accurate data [17]. Several national-level registries exist in the United States, where data for both in-hospital and out-of-hospital CA can be reported, aggregated, and analyzed [19–21]. These have increased capacity in the resuscitation QI and science fields by way of access to resources (the registries themselves) and the generation of reports for users and researchers. The design of these registries is informed by best practices and based on published standards such as the Utstein templates for resuscitation registries [22–25]. Although these design features help to ensure that the data submitted are standardized and can be rigorously analyzed, they do little to ensure that all eligible events from contributing institutions are detected and their data collected and submitted. This is especially reflected in the variability in reported incidence of IHCA and even more so with the wide range of pediatric estimates of CA [4, 5, 12]. Active and comprehensive surveillance is necessary at the individual hospital level to ensure not only both reliability and accuracy of the data being submitted but also their completeness. Without complete event detection, the true incidence of pediatric CA will be underestimated. Furthermore, survival rate estimates may be inaccurate and systematic selection biases may exist in the larger national registries. To address these weaknesses, we report the development of a semiautomated, electronic, and multidisciplinary reporting system that vastly increased our capture of the CA events.

Our integrated data collection system shows that such underestimation can be improved with a systematic approach. This institution participates in the American Heart Association's “Get With the Guidelines-Resuscitation” (GWTG-R) national registry. While reporting to this QI initiative in 2011, there were eleven detected in-hospital pediatric CA. In 2012, this number increased to fourteen of which eight occurred in the PICU, four on the general wards (floor), one in a diagnostic area (MRI), and one in the PEDS-ED. Furthermore, according to these records there were “0 pediatric events” in February, August, September, and November. When these statistics were reviewed by the members of the CPR advisory committee and compared to other institutions, these numbers appeared to underrepresent the expected frequency of events.

Using the PICU as an example, in 2011 and 2012 there were approximately 2000 admissions annually. Current reported rates of CA in PICUs are estimated to range from 1.8% to 5.5% of admissions [9, 10, 12] and therefore using these rates, the expected number of CA that should be observed in the PICU is between 36 and 110. In 2012, the observed number of events was 8 versus the expected of at least 36. This observation further aided in validating the concern that pediatric CA events were being missed using the methods of detection in place at the time.

After the implementation of the REACH surveillance system, the documented number of CA was closer to the expected incidence. First, the count of events identified only by previous methods was similar with historical counts (Table 4), suggesting that the system was not missing any that were previously being detected. Second, the difference between the CA events that would have been detected and what was detected show a true increase in events captured (e.g., 2013: 4 versus 38 events). Lastly, the calculated incidence based on the detected frequency of these events falls within the expected estimates as reported in the literature [4, 5, 9, 10, 26]. The measured incidence for PICU events (CA/admissions) was approximately 2% annually (2013: 38/2100 (1.8/100 admissions); 2014: 51/2262 (2.2/100 admissions); 2015: 54/2203 (2.5/100 admissions)), suggesting that the system was approaching 100% capture in the PICU.

The increase in detected events for the entire pediatric population by year and in previously underreported care areas indicates that the system objective to identify every pediatric CA is on its way to being met. By leveraging additional electronic sources to identify candidate events and along with a multidisciplinary team to verify CA status created a more effective system. Moreover, as comprehensive local surveillance processes or systems such as the REACH system are deployed, more accurate and reliable pediatric CA event datasets, with less selection bias, can be submitted to national registries.

Detection of every event not only promotes the completeness, accuracy, and reliability of registry data, it also provides for more opportunities to critically and objectively debrief these events. Technological advances in recent years have allowed for (1) patient and CPR performance data capture during cardiopulmonary arrest, (2) real-time feedback, and (3) postevent evaluation of the health-care provider performance. A systematic review of these technologies suggests that their use during training helps to improve skill retention [27]. Despite the ability for these devices to provide feedback during CPR, it is unclear whether this alone is sufficient to affect the sustained health-care provider performance [28–30]. Data collected during CPR have been shown to help improve subsequent CPR quality performance when used during cold debriefings (where individuals or teams are provided with feedback sometime after the event) [28, 31, 32]. Couper and Perkins assert that objective performance data are a key requirement for cold debriefing [33] and this is particularly important for determining guideline compliance, identifying poor performance, understanding high performance, and a shared mental model of what is exquisite CPR. Furthermore, these debriefings can provide opportunities to confirm the accuracy of data that will be entered in the GWTG-R registry, as medical records are often incomplete or inaccurate [34]. In 2014 and 2015, although not every event's bedside monitor data was captured, (2014: 51/102 (50%); 2015: 65/123 (53%)) the ability to use the multidisciplinary team to confirm or validate various objective measures (e.g., initial rhythm, time of pulselessness, time to initiation of CPR, time from shockable rhythm to defibrillation, and use of a device to confirm endotracheal tube placement) allowed for appropriate identification of each CA event and accurate registry reporting. Siems et al. recently confirmed the high-degree of inaccuracy in documentation of the time to initiation of CPR metric [35]. Bedside data are either lost due to the patient being discharged from the monitor system, time passing and data being overwritten, or system limitations (e.g., the OR monitor system does not interface with the data collection tool used in other care areas). Highlighting the importance of this data during debriefing has motivated leadership within each care area to implement processes to ensure that the data are not discharged or deleted prior to their collection. With the addition of a project coordinator and two volunteer clinical staff to provide capacity to collect the monitor data outside daytime hours and on the weekends, the proportion of all events with bedside data captured in 2016 has increased to 72%.

Although this system has increased the detection of events across the pediatric care areas, particularly in acute and critical care settings, it is still challenging to know if every event is being detected. More work is needed to collect appropriate denominator data for all care areas to effectively analyze CA incidence for comparison against similar institutions. Data mining of the EHR also holds the potential to determine the missed event rate, or to generate live-time alerting of CA events to be used in conjunction with or supplemental to a surveillance system such as this.

## 6. Conclusion

The implementation of a surveillance system to identify pediatric CA events, using organizationally available notification sources has resulted in an increase in CA event detection. Prior to the development of the REACH system, utmost 14 events per year were detected. After deployment, this has increased almost 9-fold to 123 pediatric CAs per year which is closer to the expected incidence. Had the system not been implemented and using predeployment methods of detection, 100% of PEDS-OR events would have been missed along with 50–70% of events from the PICU, NICU, and ED. Improved data capture through the REACH surveillance system not only provided objective assessment of guideline compliance and level of CPR quality and potential subsequent performance improvements, but they also allowed for confirmation of key data elements submitted to GWTG-R thus improving accuracy and reliability of the overall registry. An effective surveillance system, objective performance data, and active quality improvement initiatives can drive efforts to further improvements in the quality of CPR provided to children everywhere.

## Figures and Tables

**Table 1:** Counts and percentages of aggregate event, notifications, and cardiac arrest by care area.

Care area	Notifications (*n*=2,986)	Events (*n*=2,145)	Cardiac arrest (*n*=317)
Clinic	42	35	0
	(1%)	(2%)	(0%)
Floor	1,030	512	9
	(34%)	(24%)	(3%)
Imaging-diagnostic	43	19	7
	(1%)	(1%)	(2%)
NICU	225	170	52
	(8%)	(8%)	(16%)
Non-children's hospital	51	33	5
	(2%)	(2%)	(2%)
Other children's hospital	50	40	8
	(2%)	(2%)	(3%)
PACU	113	85	3
	(4%)	(4%)	(1%)
PEDS-ED	365	315	64
	(12%)	(15%)	(20%)
PEDS-OR	84	82	26
	(3%)	(4%)	(8%)
PICU	983	854	143
	(33%)	(40%)	(45%)

% values are percent of column totals.

**Table 2:** Proportion of notifications and events that are CA-related by care area.

Care area	Notifications that are cardiac arrest-related/notifications (%)	Events that are cardiac arrest-related/events (%)
Clinic	0/42	0/35
	(0%)	(0%)
Floor	32/1030	9/512
	(3%)	(2%)
Imaging-diagnostic	17/43	7/19
	(40%)	(37%)
NICU	96/225	52/170
	(43%)	(31%)
Non-children's hospital	11/51	5/33
	(22%)	(15%)
Other children's hospitals	8/50	8/40
	(16%)	(20%)
PACU	6/113	3/85
	(5%)	(4%)
PEDS-ED	80/365	64/315
	(22%)	(20%)
PEDS-OR	27/84	26/82
	(32%)	(32%)
PICU	216/983	143/854
	(22%)	(17%)
Total	493/2986	317/2145
	(17%)	(15%)

**Table 3:** Notifications per event by care area and year, stratified by cardiac arrest status.

	2013	2014	2015	Total
*Cardiac arrest event*				
Clinic	—	—	—	—
Floor	2.5	—	**4.4**	3.6
Imaging-diagnostic	2.3	—	2.5	2.4
NICU	**1.5**	1.8	**2.1**	1.8
Non-children's hospital	3.0	1.0	1.0	2.2
Other children's hospitals	1.0	1.0	1.0	1.0
PACU	—	2.0	—	2.0
PEDS-ED	1.2	1.3	1.3	1.3
PEDS-OR	1.0	1.0	1.1	1.0
PICU	**1.3**	**1.4**	**1.7**	1.5
Total	1.4	1.4	**1.8**	**1.6**
*Noncardiac arrest event*				
Clinic	1.4	1.2	1.0	1.2
Floor	1.8	2.0	**2.1**	2.0
Imaging-diagnostic	1.0	2.1	2.5	2.2
NICU	**1.1**	1.2	**1.0**	1.1
Non-children's hospital	1.6	1.4	1.3	1.4
Other children's hospitals	1.2	1.8	1.1	1.3
PACU	1.3	1.5	1.1	1.3
PEDS-ED	1.1	1.1	1.2	1.1
PEDS-OR	1.1	1.0	1.0	1.0
PICU	**1.0**	**1.1**	**1.1**	1.1
Total	1.3	1.4	**1.4**	**1.4**

Bold indicates statistically significant difference between the value for the care area and year and its complement in the comparison of cardiac arrest status group.

**Table 4:** Proportion of CA events detected only via implementation of the REACH surveillance system.

Care area	2013	2014	2015	Total
Clinic	—	—	—	—
Floor	0/4	—	0/5	0/9
	(0%)		(0%)	(0%)
Imaging-diagnostic	0/3	—	0/4	0/7
	(0%)		(0%)	(0%)
NICU	10/13	8/15	8/24	26/52
	(77%)	(53%)	(33%)	(50%)
Non-children's hospital	1/3	1/1	1/1	3/5
	(33%)	(100%)	(100%)	(60%)
Other children's hospitals	2/3	2/2	3/3	**7/8**
	(67%)	(100%)	(100%)	**(88%)**
PACU	—	1/3	—	1/3
		(33%)		(33%)
PEDS-ED	16/23	9/18	6/23	31/64
	(70%)	(50%)	(26%)	(48%)
PEDS-OR	**5/5**	**12/12**	**9/9**	**26/26**
	**(100%)**	**(100%)**	**(100%)**	**(100%)**
PICU	**34/38**	**40/51**	**28/54**	**102/143**
	**(89%)**	**(78%)**	**(52%)**	**(71%)**
Total	68/92	73/102	55/123	196/317
	(74%)	(72%)	(45%)	(62%)

## Appendix

### Development and Configuration of System Components

The following describes what configuration or development was needed to integrate each notification source into the REACH system.

#### A.1. Development: Notification Sources

- PING (Johns Hopkins, Baltimore). This organizationally developed web-based messaging service is used to communicate throughout the Children's Center and was harnessed for two particular classes of events that had the potential to represent a cardiac arrest. Integration of this system was achieved by the list owners (clinicians) adding the REACH email address to the recipient list for the following situations involving critically ill children:
- Pediatric Expedited Transfer (PET). When a critically ill child needs an expedited transfer from the Emergency Department to the PICU, a PET team is paged.
- Code Blue Button. Connexall (Connexall USA Inc., Boulder, CO). The intended use of these buttons is to summon additional help for care providers during urgent or emergency situations. When used in conjunction with a clinical telephony solution (Ascom North America, Morrisville, NC), this system allows for text-based messaging to groups of care team members through integrated call systems, clinical phones, or email. Integration was achieved by (the clinical engineer department) adding a rule that triggers an email to the REACH account when buttons are pressed.
- Emergency Response Paging. SDC Intellidesk (SDC Solutions, Manchester, NH) When the Pediatric Rapid Response Team (RRT) is called for and dispatched (at this institution for both cardiac arrest and noncardiac arrest emergencies), a message is sent via page to members of the team. The paging system can also send a copy via email. Integration was achieved by (the pager administration office) adding the REACH email address to the RRT recipient list.
- Electronic Health Record (EHR). Sunrise Clinical Manager (Allscripts, Chicago, IL). This EHR has the capability to trigger an email when cardiac arrest or rapid response flowsheets are opened and used to document the event. Integration was achieved by (a Clinical Information System technical lead) adding a rule defining the REACH email address as the recipient.
- Respiratory Therapy Services Billing. As part of the ongoing finance and accounting processes by the hospital's Respiratory Therapy (RT) organizational unit, a report of all billable services provided by respiratory therapists is automatically generated daily. This report contains codes for Respiratory Therapy services provided to patients. Three specific codes associated with CPR are often used: *Cardiac Arrest, Intubation Assist, and Manual Ventilation.* This report is sent from the billing system as a comma-separated values (CSVs) file to the RT manager. The manager created an email inbox rule to forward this to the REACH email address.
- Virtual Code Blue (VCB) Button. Prior to the development of the VCB button, when no notification source was activated, but a member of the MDQI group knew of a pediatric cardiac arrest, a manual entry would be made via the User Interface by a system administrator. The VCB notification allows for the same functionality through a secure web-based form and by any member of the organization. This form creates a notification and inserts it directly into the database, while simultaneously initiating the data collection process. This was developed as part of the System Logic and User Interface application.

#### A.2. Development: Organizational Enterprise Email

Microsoft Exchange Server (Microsoft, Redmond, WA) is this institution's enterprise email system. No custom development or server-level modifications were necessary. A dedicated email account was created, which required a formal request to system administrators. A feature known as “inbox rules” allows for an end-user to define a number of automated tasks to be performed on messages as they arrive. This feature was leveraged to identify organizational IT system-generated notifications and forward these to the MDQI listserv. Determining valid emails consisted of one or more conditions based on the sender email address and known keywords being satisfied.

#### A.3. Development: Database

The database solution used is Microsoft SQL Server Express Edition (64-bit) Version 10. It consists of 20 tables that provide data storage and relationship definitions. These provide storage and structure to standardize all IT systems notification data from free-text message formats to that which allows for the creation of relationships between notifications, events (including type and location), and collected smart defibrillator and bedside monitor data.

#### A.4. Development: System Logic and User Interface

The System Logic and User Interface were developed using ASP.NET 4.5 Web Forms (C#) and runs on Internet Information Services (IIS) version 7.5. This application requires an SSL connection, using a SHA-256, RSA (2048 bit) certificate. This application is accessible only while on the institutional network or connected remotely through a secure VPN connection, using a fully qualified domain name managed by the institutional DNS. Authentication is also managed using the institutional single sign-on (SSO) service, whereas authorization is managed by the system database. Web forms were developed to provide for management of notification, event, and smart defibrillator and bedside monitor data, as well as data export and analysis.

#### A.5. Development: Listserv

The listserv is provided as an institutional communication tool powered by Sympa (RENATER. Paris, France). This tool is configured such that only the REACH email service and list members can send to it, and that any reply is a reply-all.

#### A.6. Development: Notification Polling Service

The Notification Polling Service is a Windows Service Application, developed in C# and running on an institutional server. This application uses the Exchange Web Services Managed API v2.0 (Microsoft, Redmond, WA) to interact with the REACH mailbox and a data access library providing database access. This service checks for new messages every 10 seconds and manages previous interactions with the system through the preexisting “read-unread” built-in feature of Exchange as well as a check of the message's GUID presence in the database. This allows for MS Exchange, the database, or the polling service to be offline for any number of reasons, but resume normal operation (particularly processing of notifications and creation of events) as soon as all are available.
